# The Immune Syntax Revisited: Opening New Windows on Language Evolution

**DOI:** 10.3389/fnmol.2015.00084

**Published:** 2016-01-11

**Authors:** Antonio Benítez-Burraco, Juan Uriagereka

**Affiliations:** ^1^Department of Spanish Philology and its Didactics, University of HuelvaHuelva, Spain; ^2^Department of Linguistics, University of MarylandCollege Park, MD, USA

**Keywords:** language evolution, HGT, microbiome, immune system, brain, skull, globularity, externalization

## Abstract

Recent research has added new dimensions to our understanding of classical evolution, according to which evolutionary novelties result from gene mutations inherited from parents to offspring. Language is surely one such novelty. Together with specific changes in our genome and epigenome, we suggest that two other (related) mechanisms may have contributed to the brain rewiring underlying human cognitive evolution and, specifically, the changes in brain connectivity that prompted the emergence of our species-specific linguistic abilities: the horizontal transfer of genetic material by viral and non-viral vectors and the brain/immune system crosstalk (more generally, the dialogue between the microbiota, the immune system, and the brain).

## Introduction

Hauser et al. (2002) hypothesized that our Faculty of Language may be decomposed into a core computational system (the narrow faculty of language or narrow syntax, NS) and two interface devices; a conceptual-intentional system and an externalization system. In their view, only the NS was specific to humans. The emergence of NS is thought to be bound to a brain rewiring that gave rise to a domain-general computational ability, either a recursive capability (Hauser et al., 2002), or one for combining conceptual units that belong to distinct “core knowledge systems”, presumably linked to the changes that brought about our globular brain and braincase (Spelke, 1994, 2000, 2003; Boeckx, 2010; Boeckx and Benítez-Burraco, 2014a). The core combinatorial operation in natural language is usually called “Merge” (Chomsky, 1995). As pointed out by Boeckx and Benítez-Burraco (2014a: 5), merge allows for “the compositional, freely combining, systematic, cross-modular semantics that is manifest in all human languages”.

The retrieval of archaic human genetic materials (Green et al., 2010; McLean et al., 2011; Meyer et al., 2012; Gokhman et al., 2014; Prüfer et al., 2014; Pääbo, 2014) has uncovered genetic and epigenetic changes that may have contributed to the emergence of our species-specific mode of cognition, including our linguistic abilities (see Boeckx and Benítez-Burraco, 2014a,b; Benítez-Burraco and Boeckx, 2015). However, other changes plausibly contributed to this emergence. Recent research has shown in particular that RNA regulation of epigenetic processes, RNA editing, and the controlled mobilization of transposable elements may explain crucial aspects of the evolution of the human brain and of human cognition (Barry and Mattick, 2012).

Piattelli-Palmarini and Uriagereka (2004: p. 341) suggested that an evolutionary understanding of our NS should “link that kind of syntax with the workings of very elementary levels of biological organization, such as the structure of the adaptive immune system and its biochemical base”. According to them, “the evolution of an entire mechanism (such as NS) which establishes one or more interfaces is most likely epigenetic in nature, and viral interactions, generally understood, provide the right level of complexity” (p. 359). Appealing to viral infection built on four facts: (i) viral infection may resemble epigenetic modifications of the DNA (in contrast, gene changes are usually either too specific or too general); (ii) viral infections are commonly tissue-specific (while gene mutations are usually pleiotropic); (iii) viruses can infect an entire population (whereas gene mutations spread slowly), thus providing an approach to how language may be inherited by a group, not just an individual; and (iv) lastly, viruses can integrate into a host genome (consequently, modifications brought about by the viral DNA itself or by the integration event can be subsequently inherited by the offspring).

We will revisit this hypothesis in light of findings affecting three factors: (i) there is an intimate crosstalk between the immune system and the brain (Ziemssen and Kern, 2007; Kokaia et al., 2012; Ransohoff and Brown, 2012); (ii) horizontal gene transfer (HGT), including viral transfer, occurs in metazoans on a previously unsuspected scale (Crisp et al., 2015); this represents a form of genetic variation that affects the evolution of species (Koonin and Wolf, 2012; Syvanen, 2012; Baltrus, 2013); and (iii) the tight association between the human body and its microbiota may affect brain development, function, and evolution. We expect these factors to have reshaped the primate brain responsible for modern cognition, contributing to the emergence of language.

Recent research has shown how bacterial colonization of the gut is central to postnatal development of systems that influence brain programming and signaling, particularly the immune system (Borre et al., 2014). For example, the developing serotonergic system responds differentially to diverse microbial colonization patterns because the gut microbiota reduce the amount of tryptophan available for serotonin synthesis (O’Mahony et al., 2015). While the involved enzymes are immune-responsive (O’Mahony et al., 2015), serotonin contributes to innate and adaptive responses of the immune system (Baganz and Blakely, 2013). Interestingly, differences in serotonin levels are a hallmark of cognitive disorders involving language deficits, like autism (Abramson et al., 1989; Hranilovic et al., 2007). Neuropsychiatric disorders may also result from the distortion of serotonin/immune conversations (Baganz and Blakely, 2013). Microbes colonizing the gut produce other neuroactive compounds, including GABA and dopamine (Wall et al., 2014), as well as molecules with neuromodulatory potential, like short chain fatty acids (Stilling et al., 2014a). Moreover, Fitzpatrick (2014) and Stilling et al. (2014a) have argued that (symbiont) microbes are epigenetic effectors, as they share most of the features of classical epigenetic mechanisms. Finally, Stilling et al. (2014b) have suggested that epigenetic mechanisms mediate host-microbe communication, resulting in changes in brain adaptation and plasticity.

**Figure 1:**
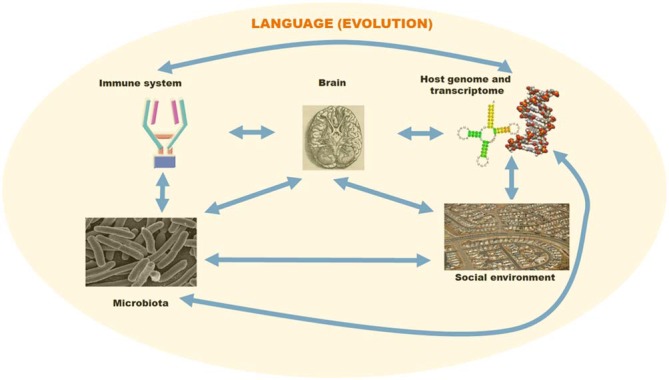
**A schematic overview of the different factors involved in language evolution**. The language faculty was brought about by changes in the primate genome and transcriptome. However, environmental cues may have contributed as well to some of the changes facilitating the emergence of modern languages. Host-pathogen interactions may have affected brain and language evolution via direct effects on brain development and indirect effects on social behavior. For this figure, pictures have been taken from Wikipedia and subsequently adapted/arranged in a composite figure.

Properties that are latent and emerge in response to a change in the environment may be relevant to the evolution of language, in view of evidence of social transmission and cultural evolution in language variation and the acquisition of grammatical properties (Boeckx, 2013; Benítez-Burraco et al., under review). While changes in the interactomes of language-related genes are a condition for “cognitive modernity”, as such they do not entail immediate “behavioral modernity”. For that, the environment arguably had to be of the right kind, exhibiting a “cultural niche” encompassing the social, behavioral, and neural conditions of human culture (see Tomasello, 2009, 2014). In a nutshell, human microbiota may be one of these conditions (Figure 1).

Obviously, we are not claiming that “a language virus” exists, or that human-specific bacterial symbionts may account for human-specific cognitive traits. What we hypothesize, instead, is that these mechanisms (e.g., a modification in our feeding habits that could have brought about a subtle change in our ancestral microbiota) may have affected brain development in some crucial sense. The complex language faculty that anatomically-modern humans (AMHs) present appears to have resulted from different kinds of changes, from mutations in key genes to alterations in the transcriptional profile of others. We expect our hypothesis to be just one piece of a large puzzle.

In section “Immune(-like) Mechanisms and language Evolution,” we discuss the convergent (co)-evolution of the brain and the immune system. We focus on the similar mechanisms that seem to account for the molecular diversity observed in both domains, but also on the crosstalk between the immune system and the brain. We then move to the realm of pathogens. In sections “Viral Vectors and Language Evolution” and “Non-Viral Vectors and Language Evolution,” we examine several genes that may have been horizontally transferred to the human genome and are expected to interact with genes that we regard as important for language evolution. In section “Microbiota and Language Evolution,” we discuss the broader effect of the microbiota on brain development and function and introduce the idea of the *language hologenome*. Finally, in section “From Brain Rewiring to Language Evolution,” we speculate on how these findings could help us improve our current understanding of the linguistic mind and its evolution.

## Immune(-Like) Mechanisms and Language Evolution

The idea that immune-related mechanisms may have contributed to the brain rewiring underlying modern syntax boils down to two facts. First, brain functions result in part from neurons assembled during development into an exponentially greater number of networks. This depends on the expression of molecular cues onto the cell surface of the neurons-to-be-assembled: tens of thousands of neuronal networks are characterized by distinctive molecular codes. Changes in brain wiring and function should be expected from changes in neural interconnection patterns relying of this complex code. The mechanisms that allow this diversity are similar to those regulating the tuning of cell receptor interactions in the immune system. Second, the immune system directly affects brain development. Consequently, we should expect changes in brain wiring and function from changes in immune response (for example, after pathogen infection).

**Figure 2:**
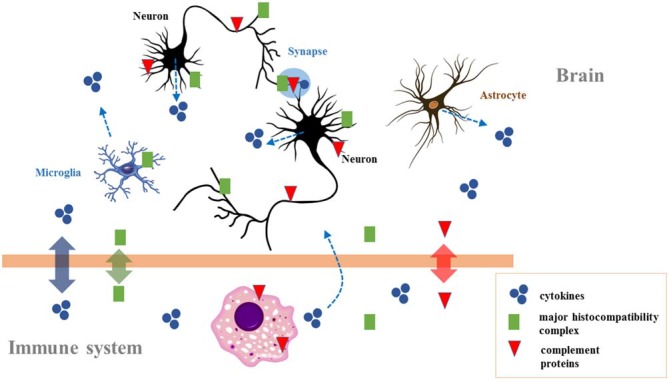
**The crosstalk between the immune system and the brain**. Immune molecules cross the blood–brain barrier on occasion of brain damage. They are also expressed during brain development, affecting neurogenesis, neuronal migration, axon guidance, and synapse formation. In the adult brain they modulate activity-dependent refinement of neural circuits and synaptic plasticity (including long-term potentiation, long-term depression, and synaptic scaling), as well as brain function (including cognition). Because chronic changes in the immune molecules levels are observed in neurodevelopmental disorders (particularly autism and schizophrenia), they could also contribute to the neurocognitive profile of affected individuals. Adapted from Garay and McAllister (2010; Figure 3).

Both parallelisms are worth exploring vis-à-vis language evolution (Figures 1, 2). Interestingly, brain and immune complexity appear to have evolved in parallel. Insects have minimal brains and no adaptive immunity, reptiles have larger brains and a basic adaptive immunity, mammals have the largest brains and full adaptive immune systems. Both systems may have evolved in a convergent fashion, if the requirements for complex intercellular communication networks ended up selecting for similarly structured networks in the immune system and our brains. Then again, actual co-evolution between the immune system and the brain may have taken place instead. We would like to highlight two aspects of these broad parallelisms.

First, many neuronal cell surface receptors involved in neuronal interactions contain immunoglobulin domains. These molecules regulate neuronal migration and survival, axon guidance, and synaptic targeting during development (Maness and Schachner, 2007). Among those in the immunoglobulin superfamily that are relevant to language one finds the ROBO/SLITs signaling proteins (see Boeckx and Benítez-Burraco, 2014b). Another interesting example is NCAM, which interacts with VCAM1, a protein bearing a fixed change (D414G) in AMHs as compared to Neanderthals/Denisovans (Pääbo, 2014, Table S1). NCAM plays a role in axonal/dendritic growth and synaptic plasticity, and ultimately the development of cognitive abilities (Prag et al., 2002; Hansen et al., 2008). Aberrant expression patterns of *NCAM* or incorrect posttranslational modifications of the protein have been linked to cognitive disorders involving schizophrenia-like symptoms (Vawter et al., 2001; Atz et al., 2007). The gene is a target of RUNX2 (Kuhlwilm et al., 2013) and FOXP2 (Spiteri et al., 2007), both crucial factors involved in language development (Boeckx and Benítez-Burraco, 2014a,b).

Before reviewing another aspect of the parallelisms, note that other surface molecules involved in cell-recognition/adhesion in brain development/functioning are often endowed with immunoglobulin-like properties. We expect common molecular mechanisms explaining diversity in immunoglobulins and neuronal adhesion molecules. For example, the functional heterogeneity of these surface molecules amounts to the independent stochastic expression of given genes’ autosomal alleles. One instance is *DSCAM*, a candidate for Down syndrome defects involved in neural wiring and innate immunity (Schmucker and Chen, 2009). Another, clustered protocadherins (Pcdhs), which are arranged in clusters and expressed by alternative promoter choice. Pcdh isoforms are further arranged in heteromultimeres that represent selective binding units for cell-cell interactions (Yagi, 2012, 2013; Sotomayor et al., 2014). Overall, clustered Pcdhs play critical roles in axonal projection, synaptic formation, dendritic arborization, and neuronal survival (Chen and Maniatis, 2013; Hirayama and Yagi, 2013). Some of these have been related to neurological diseases and cognitive disorders involving language deficits (for review, see Hirabayashi and Yagi, 2014). Thus *PCDH10* has been linked to autism (Tsai et al., 2012), while mutations in *PCDH19* cause epilepsy and mental retardation (Dibbens et al., 2008). More to our point, Williams et al. (2006), Chance and Crow (2007), or Crow (2008) argue that different chromosomal reorganizations/mutations affecting *PCDH11* triggered a modification of the brain lateralization pattern that contributed to language emergence. Finally, the editing enzymes may also explain diversity in immunoglobulin and neuronal adhesion molecules, which could affect language evolution (see Dong et al., 2012 on the downregulation of *APOBEC3A* and *APOBEC3C* in the inferior parietal lobule of psychotics; for LINE-1 and Alu elements, immobilized by these enzymes, as linked to cognitive disorders, see Muotri et al., 2010; Hancks and Kazazian, 2012; Thomas et al., 2012).

As a second aspect of crosstalk between the immune system and the brain (and, for us, the parallelism between immunity and syntax), note that immunoglobulin cell surface receptors are also active in the brain (Figure 2). They play a role in the development of different cell types (Nakahara et al., 2003) and contribute to the functional establishment in different brain areas (Andoh and Kuraishi, 2004; Nakamura et al., 2007). Moreover, their aberrant activation contributes to the pathogenesis of neurodegenerative conditions (Okun et al., 2010). For example, in amyotrophic lateral sclerosis immunoglobulin G (IgG) uptake by motor neurons affects transmitter release from motor axon terminals (Mohamed et al., 2002). More generally, most of the molecules whose production is triggered upon infection play a role in the normal development of the brain. Thus cytokines modulate neurogenesis in response to an activated immune system and seem involved in neurobiological mechanisms associated with cognitive processes (Borsini et al., 2015; Figure 2). At the same time, the altered homeostasis of cytokines impacts brain development and predisposes to mental diseases (Ratnayake et al., 2013). Ge et al. (2014) link cytokine levels, functional polymorphisms of immunity-related proteins, and language impairment. Similarly, the *complement*, a component of the defense cascade of innate immunity comprising fluid-phase and cell-associated proteins (Wagner and Frank, 2010), also plays a role in brain wiring and function (Figure 2). This “complement activation” contributes to the remodeling of synaptic circuits during early stages of brain development (Eggleton et al., 2000; Bialas and Stevens, 2013), but it is necessary as well for brain wiring after birth, a failure in which may result in autism or schizophrenia (Patterson, 2011). Overall, normal brain-immune communication is crucial for the development of the brain, while alterations in brain-immune communication (e.g., caused by pathogen infections) give rise to neuropsychiatric disorders. This is, in short, why we expect changes in brain-immune crosstalk to have contributed to brain rewiring during recent hominin evolution.

Since the logic of the “immune syntax” hypothesis was “based on properties of immune responses to viral intruders” (Piattelli-Palmarini and Uriagereka, 2004: p. 366), we next explore the putative effects of pathogenic infections on language evolution. Again, infections can affect a population, thus contributing to spread innovations, and because microbes can integrate into the host genome, modifications brought about by their genetic material or the integration event can be inherited by the offspring.

## Viral Vectors and Language Evolution

Chronic and neuropathic viral infections produce loss of neurons and axons that result in neurodegenerative and neurocognitive diseases (Karim et al., 2014). Viral infection has been hypothesized to (subtly) contribute, also, to cognitive developmental disorders. According to Fruntes and Limosin (2008), prenatal exposure to viruses may cause early brain damage and an aberrant pattern of neuronal migration and expression of neural cell adhesion molecules that may result in schizophrenia. Some viral infections can give rise to loss of language (Marques et al., 2014). Viruses are hypothesized to cause this effect by direct brain lesion, by triggering an autoimmune response during development, or by inducing the maternal immune activation during growth (Garbett et al., 2012). Interestingly, viruses that specifically attack the immune system can produce cognitive impairment too, as commonly observed in HIV-infected patients (Rosca et al., 2012). Viral infection can also affect the cellular mechanisms involved in somatic variation and neuronal diversity within the brain (with deleterious consequences). For instance, changes in the splicing profile of *MOR* induced by the HIV-1 virus sometimes result in neurocognitive impairment (Dever et al., 2012).

Importantly, viruses are also able to transfer DNA or RNA fragments to the host species that may be permanently integrated in their genomes and be subsequently transmitted to offspring (Liu et al., 2011). The human genome expresses multiple genes acquired from or potentially transferred by viruses (Crisp et al., 2015). A literature search [via PubMed and OMIM (http://www.ncbi.nlm.nih.gov/omim/)] helped us determine whether, and if so to what extent, some of these genes may have contributed to the changes that we believe important for language evolution (reviewed in Boeckx and Benítez-Burraco, 2014a,b; Benítez-Burraco and Boeckx, 2015). We have relied as well on computer tools [mostly on String 10 (http://string-db.org/)] to learn about the robustness of the links we posit. As for phylogenetic changes, we have relied on available data on genetic and epigenetic changes that occurred after our split from Neanderthals and Denisovans.

**Figure 3:**
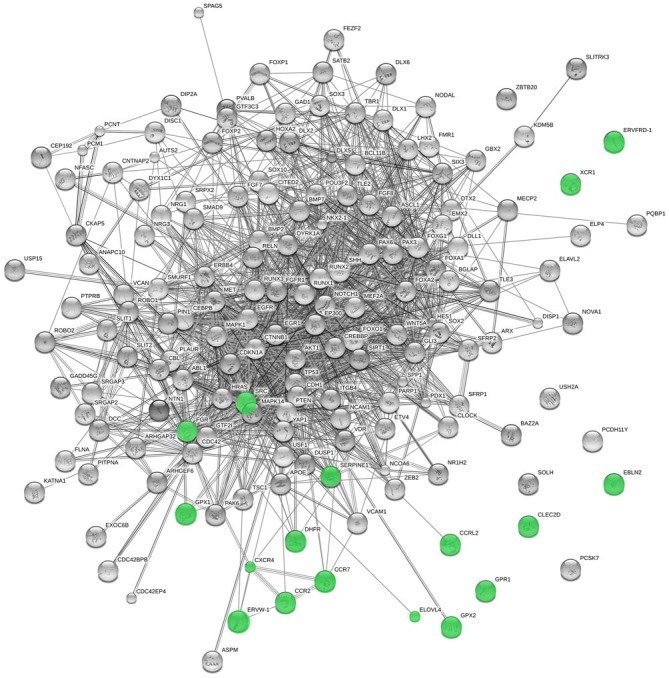
**Functional links predicted by String 10 among candidates for the evolution of language (nodes in gray) and the horizontally-transferred genes from viruses highlighted here as potential new candidates (nodes in green)**. Stronger associations between proteins are represented by thicker lines. The medium confidence value was 0.0400 [a 40% probability that a predicted link exists between two enzymes in the same metabolic map in the KEGG database (http://www.genome.jp/kegg/pathway.html)]. String 10 predicts associations between proteins that derive from a limited set of databases: genomic context, high-throughput experiments, conserved coexpression, and the knowledge previously gained from text mining (Szklarczyk et al., 2015). This is why the figure does not represent a fully connected graph (evidence for additional links are provided in the main text). Importantly, the diagram only represents the potential connectivity between the involved proteins, which has to be mapped onto particular biochemical networks, signaling pathways, cellular properties, aspects of neuronal function, or cell-types of interest that can be confidently related to aspects of language development and function (see Table 1).

We have found that several of the genes potentially transferred from viruses are candidates for cognitive disorders entailing language deficits, or play a role in aspects of brain development and function that we believe relevant for language processing, or interact with some of our candidates for language evolution (Table 1; Figure 3). Among them, we have found genes that are upregulated upon RUNX2 transfection [*SERPINE1, ELOVL4, CXCR4, CCR7, GPX2, GPR1*, and *DHFR* (Kuhlwilm et al., 2013)], and several targets of *FOXP2* [*FGR, CLEC2D, CCRL2, CXCR4*, and *GPR1* (Spiteri et al., 2007)]. *RUNX2* is our core candidate underlying the changes that prompted the globularization of the human brain, whereas *FOXP2* is a renowned gene involved in vocal learning (Graham and Fisher, 2013). The evolutionary modification of the interactomes of both genes may have contributed to the emergence of our language-readiness and refined the devices involved in the externalization of language (for details, see Boeckx and Benítez-Burraco, 2014a,b; Benítez-Burraco and Boeckx, 2015).

**Table 1:** **Genes discussed in sections 3 and 4**.

Core candidate genes for language evolution	Top-25 GO biological processes of core candidates genes for language evolution	Viral genes important for language evolution	Non-viral genes important for language evolution
*ABL1*	Nervous system development	*CCR2*	*AHNAK*
*AKT1*	Neurogenesis	*CCR7*	*AHNAK2*
*APOE*	Generation of neurons	*CCRL2*	*AL158821.1*
*ARX*	Organ development	*CLEC2D*	*AL356585.1*
*ASCL1*	System development	*CXCR4*	*AP4E1*
*AUTS2*	Central nervous system development	*DHFR*	*ASTN2*
*BMP2*	Multicellular organismal development	*EBLN2*	*CARNS1*
*BMP7*	Anatomical structure morphogenesis	*ELOVL4*	*CDKL5*
*CBL*	Regulation of developmental process	*ERVFRD-1*	*CENPF*
*CDC42*	Cellular developmental process	*ERVW-1*	*CYP26A1*
*CEBPB*	Anatomical structure development	*FGR*	*CYP26C1*
*CITED2*	Cell differentiation	*GPR1*	*DAZ2*
*CMIP*	Regulation of multicellular organismal development	*GPX1*	*FAM230A*
*CNTNAP2*	Single-organism developmental process	*GPX2*	*FLJ22447*
*CREBBP*	Developmental process	*SERPINE1*	*GIMAP8*
*CTNNB1*	Head development	*SRC*	*GOLGB1*
*DISP1*	Cell development	*XCR1*	*HAS1*
*DLL1*	Neuron differentiation	*YES1*	*HAS3*
*DLX1*	Brain development		*IL4I1*
*DLX2*	Regulation of multicellular organismal process		*IRG1*
*DLX5*	Tissue development		*LMO7*
*DLX6*	Forebrain development		*MAP6*
*DYRK1A*	Regulation of cell differentiation		*MSRA*
*EGR1*	Regulation of cell development		*MT-ND4L*
*EP300*	Regulation of gland development		*MYO10*
*ERBB4*			*NQO1*
*FEZF2*			*PADI2*
*FMR1*			*PADI3*
*FOXG1*			*PLAU*
*FOXO1*			*RAD21-AS1*
*FOXP1*			*RIMKLA*
*FOXP2*			*RTL1*
*FOXP2*			*SERPINB2*
*GAD1*
*GBX2*
*GLI3*
*GTF2I*
*HES1*
*LHX2*
*MAPK1*
*MECP2*
*MEF2A*
*NCAM1*
*NCOA6*
*NFASC*
*NKX2–1*
*NODAL*
*NRG1*
*OTX2*
*PAX6*
*POU3F2*
*PTEN*
*ROBO1*
*ROBO2*
*RUNX2*
*SATB2*
*SHH*
*SIRT1*
*SLIT1*
*SLIT2*
*SOLH*
*SPAG5*
*SRGAP2*
*SRGAP3*
*SRPX2*
*TBR1*
*TP53*
*TSC1*
*VCAM1*
*ZBTB20*

*The first column contains core candidates for the evolution of language as posited by Boeckx and Benítez-Burraco (2014a,b) and Benítez-Burraco and Boeckx (2015). The second provides a GO classification of these genes according to Panther (http://pantherdb.org); only the top-25 functions after a Bonferroni correction have been included. The last two columns include the horizontally-transferred genes from viruses and non-viral organisms, respectively, highlighted here as potential new candidates for language evolution*.

If language evolution was affected by changes in the immune system/brain crosstalk, it is interesting that *GPR1* turns out to be among the genes potentially transferred to humans from viruses. This gene is expressed in the hippocampus of primates only (as compared to rodents) and encodes an orphan G protein-coupled receptor (Marchese et al., 1994). GPR1 functions as a co-receptor for some viruses, making the cell more susceptible to infection (Shimizu et al., 1999). This makes us wonder whether posterior infections or infection-related events affected the evolution of cognition in primates only, as compared to other mammals. We also find it interesting that many genes that were putatively transferred from viruses would encode cytokine- and chemokine-related proteins. Again, such immune-signaling molecules and their functional partners do not only mediate viral infection: they are also expressed in the brain, in whose development and function they play a role, in synaptic pruning (Paolicelli et al., 2011), brain disease (Moretti et al., 2015), and memory and learning (Williamson and Bilbo, 2013); see e.g., van der Meer et al. (2000) on *CCR2* and *CXCR4*, two of the genes in Crisp et al.’s (2015) list. It should be established whether any of these genes played a role in the evolution of the memory and learning capacities presupposed in the faculty of language.

Other transferred genes (present in primates only) are endogenous retroviruses (HERVs). These are non-coding DNA remnants of retroviral infections occurred during primate evolution (Antony et al., 2004). While HERV activity pertains to individual genomic variation associated to chromosomal rearrangements (Weckselblatt and Rudd, 2015), it may also account for evolutionary differences across species, as accumulation of retroelements parallels the increment of evolutionary complexity of the host species (Kidwell, 2002). In all vertebrates, there is evidence of transposable element-mediated genomic rearrangements potentially associated with or subsequent to speciation events, which suggests that these are drivers of genomic and biological diversity in vertebrates (Böhne et al., 2008). Interestingly, HERVs travel in waves of infection and subsequently transition from exogenous to endogenous forms (Ishida et al., 2015). Curiously, the emergence of HERVs coincided with that of adaptive immunity (Litman et al., 2010). Although the vast majority of retroviruses have been inactivated, some are still expressed in different tissues (Seifarth et al., 2005; Yi et al., 2006), playing regulatory functions and more (Bannert and Kurth, 2006). Activation or upregulation of HERVs have been linked to AMH-specific neurocognitive conditions like autism (Balestrieri et al., 2012) or schizophrenia (Frank et al., 2005), which helps us illustrate how HERVs may have affected genes involved in language evolution.

According to Suntsova et al. (2013) HERVs may serve as tissue-specific enhancers for brain-related genes involved in schizophrenia—specifically *PRODH*, which codes for a proline dehydrogenase enzyme that plays a role in neuromediator synthesis in the brain. The mechanism involves the transcription factor SOX2, regulated by RUNX2 (Yoon et al., 2014). At the same time, SOX2 regulates *PQ15P1*, a gene linked to developmental delay, intellectual disability and microcephaly (Li et al., 2013), which interacts with POU3F2 (Li et al., 2013). Intron 8 of *FOXP2* contains an AMH-specific substitution that affects a binding site for POU3F2 (Maricic et al., 2013). Moreover, schizophrenia has been claimed to result from epigenetic changes that deregulate HERV-activity (Diem et al., 2012). Importantly, among the environmental factors causing these epigenetic changes one finds viral infections, which can deregulate the epigenetic control naturally involved in silencing HERVs via the transactivation of endogenous retroviruses (Perron and Lang, 2010). We believe it is worth exploring whether any of these effects played a role in language evolution.

Among the genes that may have been transferred to primates we found *XCR1*, which encodes a chemokine receptor belonging to the G protein-coupled receptor superfamily (Heiber et al., 1995) and is a FOXP2 target (Vernes et al., 2011). Additionally, *ERVW-1* and *ERVFRD-1* are found in all primates except tarsiers. *ERVW-1* encodes syncytin 1, a membrane protein that contributes towards immune tolerance and is found upregulated in astrocytes and glial cells of individuals with multiple sclerosis (Antony et al., 2004). *ERVFRD-1* encodes syncytin 2, a membrane protein with inmunosuppresive activity (Blaise et al., 2003). Similarly, *EBLN2* (found only in macaques, gibbons, and the great apes) encodes a protein akin to the Borna disease viruses (Horie et al., 2010). One gene was transferred to our genome after our split from the great apes; namely, *AP001468.1*. Unfortunately, it encodes a protein of unknown function.

To be clear: we are not claiming that all those genes were transferred to (and spread among) modern humans by a viral vector and that these events allowed modern language to emerge and spread so quickly. Most of the genes highlighted were transferred *before* the split of great apes from other mammals. However, because some of these are functionally related to genes that show differences when comparing AMHs to Neanderthals and/or Denisovans, we shouldn’t discard differences between hominin species regarding the functions these genes contribute to. Such may be the case of *SRC*, for instance, which is functionally related to *VCAN*: the specific blockade of Src activity abolishes versican-1-induced differentiation of PC12 cells into neurons (Wu et al., 2004). Versican-1 is involved in neurite outgrowth of hippocampal neurons (Xiang et al., 2006) and shows a fixed N3042D change in AMHs (Pääbo, 2014; Table S1). Similarly, two human-specific conserved deletions (hCONDELs; although shared with Neanderthals) exist upstream *YES1* and downstream *GPX1*, respectively (McLean et al., 2011)—which are two of the genes highlighted by Crisp et al. (2015).

Most changes implicated in language evolution are expected to have impacted the transcriptome. Regulatory switching due to the horizontal transfer of regulatory regions have been attested in bacteria (Nijveen et al., 2012; Oren et al., 2014). It may be transposable elements that harbor these regulatory elements, allowing them to move across strains (Siddique et al., 2011). Also, the consequences for gene regulation of the viral infections occurred during our recent history are worth mentioning. For example, HERVs are endowed with transcriptional activity enabling them to function as alternative promoters or enhancers, ultimately to modify the expression of neighboring genes (Le Dantec et al., 2015). Additionally, viral infections can modify the brain epigenetic landscape, as HERVs illustrate. Thus, we should expect the impact of viral infections on brain function and evolution to go beyond the roles reviewed above.

Most epigenetic changes brought about by viruses affect the expression of host immune genes, to render the immune responses inactive to their antigens (Adhya and Basu, 2010). However, viral infection *per se* may induce changes in the brain epigenetic landscape even in the latent phase. For example, latent HIV-1 is associated with increased levels of BCL11B, a chromatin modifier encoded by one the genes regulated by RUNX2, which may result in abnormal transcriptomes (Kuhlwilm et al., 2013). Exogenous factors exacerbate the impact of viral infections on epigenetic modification of the brain (Desplats et al., 2013). For instance, HIV-1 infection in conjunction with drug abuse brings about changes in the expression of *DNMT1* (a key enzyme responsible for DNA methylation), which result in differential methylation on genes related to neurodegeneration and dopamine metabolism in the frontostriatal circuits. Drugs too can cause epigenetic changes at the viral promoter, resulting in altered gene expression (Shirazi et al., 2013). One has to wonder whether such mechanisms contributed to changes required for the emergence of language-readiness.

## Non-Viral Vectors and Language Evolution

**Figure 4:**
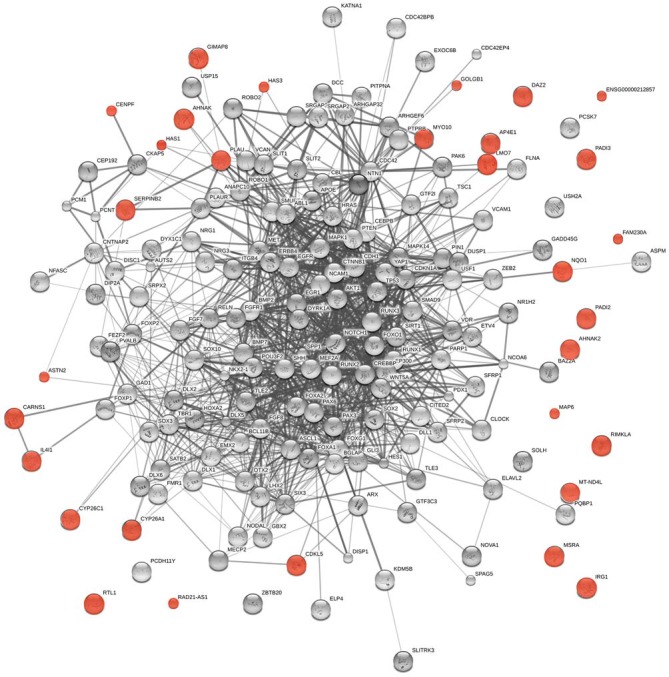
**Functional links predicted by String 10 among core candidates for language evolution (nodes in gray) and the horizontally-transferred genes from non-viral organisms highlighted here as potential new candidates for language evolution (nodes in red)**. The medium confidence value was 0.0400. The caveats noted for Figure 3 apply.

The logic of Piattelli-Palmarini and Uriagereka’s hypothesis applies to *any infectious vector* that may transfer genetic material. There is growing evidence suggesting that the microbiota impact brain development and function and, ultimately, cognition and behavior (Cryan and Dinan, 2012 and see “Introduction” Section). Nearly 150 genes from other organisms are expressed in human cells (Crisp et al., 2015). Following the *modus operandi* described in section “Viral Vectors and Language Evolution,” we have found among them language-related genes, including targets of RUNX2 (*CENPF*, *SERPINB2*, *IL4I1*, *AHNAK*, *GOLGB1*, *NQO1*, *AHNAK2*, *RTL1*, and* LMO7*) and FOXP2 (*HAS1* and *PLAU*) (Spiteri et al., 2007; Figure 4).

Other genes are functionally related to genes in the interactomes that we believe important for language evolution (Figure 4) or give rise to language or cognitive disorders entailing aberrant modes of thinking when mutated. Thus, *MAP6* encodes a protein that binds to and stabilizes microtubules and has been related to schizophrenia (Shimizu et al., 2006). In mice, deletion of *Map6* gives rise to synaptic defects and brain dysfunctions, ultimately to cognitive deficits similar to those observed in schizophrenics (Volle et al., 2013). *CDKL5* has been related to Rett-like syndrome and X-linked West syndrome, two cognitive disorders entailing language deficits (Kalscheuer et al., 2003; Tao et al., 2004; Scala et al., 2005). Mutations or deletions in *AP4E1* cause a syndrome involving microcephaly, facial dysmorphisms, cognitive impairment and speech delay (Abou Jamra et al., 2011; Moreno-De-Luca et al., 2011). *MYO10* controls the direction and morphogenesis of cells during radial cortical neuronal migration (Ju et al., 2014). Also of interest are two isoforms of the hyaluronan synthase, encoded by *HAS1* and *HAS3*. *HAS1* is upregulated in astrocytes during normal brain aging (Cargill et al., 2012), whereas *Has3*(–/–) mice exhibit altered neuronal activity and seizures (Arranz et al., 2014). Lastly, *ASTN2* regulates the levels of ASTN1, a neuron-glial ligand important for glial-guided neuronal migration, a key step in the development of laminar architecture of cortical regions of the mammalian brain (Wilson et al., 2010). Mutations in *ASTN2* have been related to neurodevelopmental disorders, including autism spectrum disorder and speech delay (Lionel et al., 2014), and to Alzheimer’s disease (AD; Wang et al., 2015a).

Several of the genes transferred from non-viral organisms are involved in the metabolism of retinoic acid in the brain. Retinoic acid controls brain changes relevant to language development, in connection with *FOXP2* (for review, see Benítez-Burraco and Boeckx, 2014), which potentiates retinoic acid induction of genes involved in neural differentiation (Devanna et al., 2014). Two of the three all-trans retinoic acid—degrading cytochromes, *CYP26A1* and *CYP26C1*, remove retinoic acid in the forebrain to ensure proper head development (Ribes et al., 2007; Uehara et al., 2007). Both genes are also involved in the regulation of retinoid signaling across multiple connected song nuclei and auditory brain regions in songbirds, modulating the circuitry that underlies the acquisition and production of learned vocalizations (Roeske et al., 2014). Another gene worth highlighting is *MSRA*, which is regulated by retinoic acid (Pascual et al., 2009) and regulates COMT activity (hence dopamine levels) in the brain (*COMT* is a strong candidate for several cognitive disorders, including schizophrenia; Moskovitz et al., 2014). Finally, among the genes transferred from bacteria we have found *IRG1*. This is an interferon-stimulated gene regulated by microRNA that reduces the susceptibility of neurons from specific areas of the brain to viral infection (Cho et al., 2013).

Although these genes were transferred between the common ancestors of Chordata and the primates, they are functionally related to genes involved in language evolution (Figure 4), some of which have changed after our split from Neanderthals and Denisovans. However, a handful were transferred later. Found in primates only, except tarsiers, *FAM230A* contains a site of frequent translocations and micro-deletions in DiGeorge syndrome (Kurahashi et al., 2007), a complex disease involving language deficits (Swillen et al., 1999; Glaser et al., 2002). *MT-ND4L* is found in great apes only and encodes the subunit 4L of the mitochondrial NADH dehydrogenase. Two genes of unknown function (*AL356585.1* and *AL158821.1*) are shared by gorillas, chimpanzees and humans, whereas two are found in chimpanzees and humans only: *DAZ2*, a member of the DAZ gene family involved in spermatogenesis, and *RAD21-AS1*, which encodes a ncRNA (*RAD21*), is highly expressed in human fetal cerebral cortex, and is thought to play a key role in brain development (Pemberton et al., 2007). Finally, *FLJ22447* was transferred to humans only, although before the split from Neanderthals/Denisovans (Gokhman et al., 2014, Table S2). This gene encodes an ncRNA that interacts with *FUS*, an RNA/DNA binding protein involved in transcription, DNA repair, and RNA splicing, which has been related to frontotemporal dementia (Wang et al., 2015b).

Some of the genes transferred from other organisms may be differentially regulated in AMHs, as compared to other close hominins, resulting from several factors. First, from human-specific deletions, like the one affecting the region upstream *PADI3* (McLean et al., 2011). Second, from differences in methylation patterns, like those expected for *MSRA*, involved in COMT action (Moskovitz et al., 2014); *PADI2*, involved in the catabolism of myelin basic protein (Asaga et al., 2002); *GIMAP8*, linked to AD (Ishigami et al., 2005) and multiple sclerosis (Mastronardi et al., 2007); *CARNS1*, which catalyzes the biosynthesis of homocarnosine (Drozak et al., 2010); and *FLJ22447* (Gokhman et al., 2014, Table S2). Third, differential regulation may result from differences across hominin species in timing of expression: this could be the case of genes regulated by MEF2A (Liu et al., 2012a), like *CDKL5* (reviewed above) and *RIMKLA*, which encodes N-acetylaspartylglutamate synthetase II, an enzyme synthesizing the neurotransmitters N-acetylaspartylglutamylglutamate and N-acetylaspartylglutamate (Neale et al., 2011). Lastly, differential regulation can arise from poorly understood interactions with the microbiome during development (not just evolution).

## Microbiota and Language Evolution

The impact of microbiota in the host (neuro)development transcends the effect of horizontally-acquired genes. Accordingly, we should expect some impact on language evolution (Figure 1). Microbiota may modulate the host transcriptome via the interaction with RNA editing enzymes (Schellekens et al., 2012) or RNA methylation enzymes (Zheng et al., 2013), its effects on histone acetylation levels (MacFabe et al., 2011) or its mimicry of the host epigenetic machinery via specific proteins (Bhavsar et al., 2007) or ncRNAs (Liu et al., 2012b). The microbial and host genomes can be regarded as an extended genome or *hologenome*, as the former complements missing components of the latter (see Rosenberg and Zilber-Rosenberg, 2014). We should thus study the genomes of our microbial endosymbionts if seeking a full account of the language gene network (or *language hologenome*). For example, changes in the gut microbiota may have allowed changes in the gastrointestinal tract that contributed to the emergence of larger brains within the primate lineage (Aiello and Wheeler, 1995). Moreover, since different microbiota are maintained by closely related species in the same environment (Franzenburg et al., 2013), cognitive differences between closely related hominin species may be partially due to the effect of differential microbiota on brain development and function. Language has a social dimension that affects its acquisition by children and perhaps some of its core properties; interestingly, changes in the social environment affect the individual microbiota and the immune response (O’Mahony et al., 2009). Importantly, recent research suggests that viruses too are part of the microbiota (Virgin, 2014).

At the same time, because they are easily transmissible, aspects of social behavior may have evolved to facilitate the transfer of beneficial microbes that protect from pathogens (Lombardo, 2008; Montiel-Castro et al., 2013, 2014). Cognitive diseases involving social dysfunction (e.g., autism) entail alterations in microbiota composition and function, as attested in humans (Ming et al., 2012) and animal models for the diseases (Hsiao et al., 2013; Desbonnet et al., 2014). There seems to also exist a critical period for the acquisition of microbiota-dependent social abilities, because some time after weaning, germ-free animals that lack social cognition are unable to achieve conspecific recognition memory even after microbiota replenishment (Desbonnet et al., 2014). As Stilling et al. (2014b: p. 11) puts it, “the microbiome represents a further interface for environmental influence and a dynamic source for transgenerational developmental regulation [M]icrobiota … accelerate short-term environmental adaptation and may be especially helpful in unifying different theories of host-microbe co-evolution and the evolution of the ‘social brain’.” In short, we expect the microbiota to account for some aspects of the “cultural niche” that allowed the transition from modern cognition to modern behavior and thus full-fledged languages.

## From Brain Rewiring to Language Evolution

What is, in the end, the connection between the “wetware” and “mindware” that results in observed behaviors, whether low-level activities of bacterial genes or the high-level accomplishments of human cognition and culture? After reminding us of the Synaptic Plasticity Hypothesis (SPH, that during memory formation, synapses undergo activity-dependent alterations), Gallistel and King (2009; p. 278 and ff.) separate (a) the role of extracting behavioral *information* from an animal’s experience from (b) the ability to *carry this information in time* in a computationally accessible format. Rejecting the idea that SPH entails synaptic conductance, they suggest that memory should be implemented “at the sub-molecular level”: “Given how much of the requisite machinery is already realized … in DNA and RNA … [It would be] … curious if a basic function that could be better implemented at the lowest possible level of structure … were found to be implemented instead at the circuit level, … requiring orders of magnitude more physical resources.” They then emphasize the speed of neural computation, particularly “given that signals travel eight orders of magnitude more slowly in the nervous system than they do in a conventional computer.” It is worth clarifying this point.

Presupposed throughout this work is the Computational Theory of Mind (CTM; Fodor, 1975, 1994, 1998): the mind can be seen as an information-processing system, “thinking” being a computational process (a logical manipulation of symbolic representations). In a system of this nature, as Gallistel and King put it: “most of the signal flow is to and fro between memory, where the symbols reside when not entering computations, and the processing machinery that implements [them]”. They then reason: “Given that signals travel slowly in neural tissue, the only way to minimize the time consumed in transferring information from memory to the processing machinery and back again is to place … memory [and] processing machinery as close together as is physically possible.” After quoting Feynman’s (1960) dictum that “There is plenty of room at the bottom,” Gallistel and King conclude that, in a neurobiological context this pushes the computational system to the realm of nucleic acid … Gallistel and King are not speaking of human cognition—they are analysing animal cognition in general. If the place to carry information forward in time within animal minds is “at the bottom”, that should be where the evolution of complex computation, of the sort presupposed in language, must have proceeded; this of course is the idea behind the “immune syntax”.

Second, it is worth noting some of the parallels that exist between the well-known properties of the immune system and NS as presently understood, as advanced by Piattelli-Palmarini and Uriagereka (2004) and see Piattelli-Palmarini and Uriagereka (2008):

1. Adaptive immunity creates immunological memory after an initial response to a specific pathogen, and leads to a response to subsequent encounters with that pathogen. Similarly, the computational system creates a lexical memory after an initial response to an acquired word, allowing for a recall to subsequent encounters with that very word.
2. There are two main classes of adaptive immune responses: antibody responses and cell mediated immune response that are also carried by two different lymphocytes (B cells and T cells). Similarly, there are two main broad classes of words: nouns and verbs, which are arguably carried by two different lexical dimensions.
3. Pathogen-specific sensors are “acquired” during the lifetime of the organism (the acquired response is said to be “adaptive” because it prepares the body’s immune system for future infections). Similarly, lexical items are acquired during the lifetime of the human organism (the acquired response could be said to be “adaptive” in that it prepares the linguistic system for future encounters with words).
4. Because of accelerated somatic mutations and irreversible genetic recombination of antigen receptor gene segments, a few genes generate a vast number of different antigen receptors. Similarly, because of Merge, a few lexical items generates a vast number of different sentences.

This may be just a case of convergent evolution, but it may be well an example of real crosstalk between the immune system and the brain. Piattelli-Palmarini and Uriagereka went into further similarities, but since presenting those would require us to delve more deeply into the nature of NS computations, (1) through (4) are enough to make the point. Coincidences or metaphors? Perhaps. During the last 150 years, neurolinguistics has attempted to map language to the brain, usefully so. Nonetheless, as Poeppel (2012) notes, mapping is not explaining. Key components of our description of language, even those as basic as memory, are still elusive. Currently, neurolinguistics is trying to distill language into a set of computational primitives (otherwise not specific to language) that form the basis for more complex representations and computations. Our speculation should be seen in that light.

One promising approach is the decomposition of language into a suite (or grammar) of endogenous brain rhythms (see Giraud and Poeppel, 2012 on speech). Brain oscillations are primitive units of brain function and are conserved across species while, at the same time, vary across disorders (Buzsáki and Watson, 2012; Buzsáki et al., 2013). Because we agree with Gallistel and King that whatever goes on “at the bottom” (nucleic interactions and more) may be closer to the CTM than the still vague associations of neurophysiology, it may worth exploring this complex parallel play between the immune system, the brain, the genome, and the microbiome.

## Conclusions

The complex evolutionary process resulting in the emergence of syntax (language in a complex sense) did not only depend on mutations occurred in particular genes, important for brain development and function. As noted by many (Enard et al., 2002; Khaitovich et al., 2006; Sikela, 2006; Vallender et al., 2008; Varki et al., 2008), most such mutations probably affected the transcriptome, involving changes in the epigenetic landscape of the primate brain. In this article, we have built on a hypothesis put forth by Piattelli-Palmarini and Uriagereka, “the immune syntax”, to explore another source of variation that may have contributed to the emergence of modern cognition and language: host-pathogen(-like) interactions occurred during evolution.

Because of the growing interest in genes potentially involved in language evolution, we have focused on genes that may have been transferred by other organisms to the human genome (though other mechanisms may have played a role). Literature-based assembly of gene-to-gene interactions (and their evolutionary consequences) has limitations. The links we have highlighted must be experimentally tested in ways that we ourselves cannot undertake, in order to prove their putative biological meaningfulness regarding brain development and cognitive evolution. Some sources of evidence we have used are stronger than others (e.g., data on direct protein to protein interactions as compared to data on genes that are up- or down-regulated after gene transfection). We also expect the vast literature and datasets to be selectively biased as they focus on given genes, processes, or different methods of interest. As a consequence, the genes we have highlighted should be regarded as mere *candidates for future research*.

We believe the links reviewed are robust in light of our current knowledge of the biological underpinnings of the language faculty, and meaningful for research on its evolution. For all its admitted limitations, our research may offer signposts for the future of this topic, at a stage of research in cognitive biology that is early enough to be fascinating, but hopefully not too early to be reckless.

## Author Contributions

AB-B explored the molecular and physiological links between the immune system and the brain, gathered the genetic data, and established the relevance of the genes under discussion for language evolution. JU authored the original hypothesis and reinterpreted it according to the biological evidence.

## Conflict of Interest Statement

The authors declare that the research was conducted in the absence of any commercial or financial relationships that could be construed as a potential conflict of interest.

## References

[B1] Abou Jamra R. Philippe O. Raas-Rothschild A. Eck S. H. Graf E. Buchert R.. (2011). Adaptor protein complex 4 deficiency causes severe autosomal-recessive intellectual disability, progressive spastic paraplegia, shy character and short stature. Am. J. Hum. Genet. 88, 788–795. 10.1016/j.ajhg.2011.04.019 21620353 PMC3113253

[B2] Abramson R. K. Wright H. H. Carpenter R. Brennan W. Lumpuy O. Cole E.. (1989). Elevated blood serotonin in autistic probands and their first-degree relatives. J. Autism Dev. Disord. 19, 397–407. 10.1007/bf02212938 2793785

[B3] Adhya D. Basu A. (2010). Epigenetic modulation of host: new insights into immune evasion by viruses. J. Biosci. 35, 647–663. 10.1007/s12038-010-0072-9 21289446

[B4] Aiello L. C. Wheeler P. (1995). The expensive-tissue hypothesis: the brain and the digestive system in human and primate evolution. Curr. Anthropol. 36, 199–221. 10.1086/204350

[B5] Andoh T. Kuraishi Y. (2004). Primary sensory neurons express the high affinity IgG Fc gamma RI receptor and responds to IgG-antigen complex. J. Pharmacol. Sci. 94:74P 10.1096/fj.02-1169fje

[B6] Antony J. M. van Marle G. Opii W. Butterfield D. A. Mallet F. Yong V. W.. (2004). Human endogenous retrovirus glycoprotein-mediated induction of redox reactants causes oligodendrocyte death and demyelination. Nat. Neurosci. 7, 1088–1095. 10.1038/nn1319 15452578

[B7] Arranz A. M. Perkins K. L. Irie F. Lewis D. P. Hrabe J. Xiao F.. (2014). Hyaluronan deficiency due to Has3 knock-out causes altered neuronal activity and seizures via reduction in brain extracellular space. J. Neurosci. 34, 6164–6176. 10.1523/JNEUROSCI.3458-13.2014 24790187 PMC4004806

[B8] Asaga H. Akiyama K. Ohsawa T. Ishigami A. (2002). Increased and type II-specific expression of peptidylarginine deiminase in activated microglia but not hyperplastic astrocytes following kainic acid-evoked neurodegeneration in the rat brain. Neurosci. Lett. 326, 129–132. 10.1016/s0304-3940(02)00334-8 12057845

[B9] Atz M. E. Rollins B. Vawter M. P. (2007). NCAM1 association study of bipolar disorder and schizophrenia: polymorphisms and alternatively spliced isoforms lead to similarities and differences. Psychiatr. Genet. 17, 55–67. 10.1097/ypg.0b013e328012d850 17413444 PMC2077086

[B10] Baganz N. L. Blakely R. D. (2013). A dialogue between the immune system and brain, spoken in the language of serotonin. ACS Chem. Neurosci. 4, 48–63. 10.1021/cn300186b 23336044 PMC3547518

[B11] Balestrieri E. Arpino C. Matteucci C. Sorrentino R. Pica F. Alessandrelli R.. (2012). HERVs expression in Autism Spectrum Disorders. PLoS One 7:e48831. 10.1371/journal.pone.0048831 23155411 PMC3498248

[B12] Baltrus D. A. (2013). Exploring the costs of horizontal gene transfer. Trends Ecol. Evol. 28, 489–495. 10.1016/j.tree.2013.04.002 23706556

[B184] Bannert N. Kurth R. (2006). The evolutionary dynamics of human endogenous retroviral families. Annu. Rev. Genomics Hum. Genet. 7, 149–173. 16722807 10.1146/annurev.genom.7.080505.115700

[B13] Barry G. Mattick J. S. (2012). The role of regulatory RNA in cognitive evolution. Trends Cogn. Sci. 16, 497–503. 10.1016/j.tics.2012.08.007 22940578

[B189] Benítez-Burraco A. Boeckx C. (2014). FOXP2, retinoic acid and language: a promising direction. Front. Cell Neurosci. 8:387 10.3389/fncel.2014.00387 25431551 PMC4230053

[B14] Benítez-Burraco A. Boeckx C. (2015). Possible functional links among brain- and skull-related genes selected in modern humans. Front. Psychol. 6:794. 10.3389/fpsyg.2015.00794 26136701 PMC4468360

[B16] Bhavsar A. P. Guttman J. A. Finlay B. B. (2007). Manipulation of host-cell pathways by bacterial pathogens. Nature 449, 827–834. 10.1038/nature06247 17943119

[B18] Blaise S. de Parseval N. Bénit L. Heidmann T. (2003). Genomewide screening for fusogenic human endogenous retrovirus envelopes identifies syncytin 2, a gene conserved on primate evolution. Proc. Natl. Acad. Sci. U S A 100, 13013–13018. 10.1073/pnas.2132646100 14557543 PMC240736

[B17] Bialas A. R. Stevens B. (2013). TGF-β signaling regulates neuronal C1q expression and developmental synaptic refinement. Nat. Neurosci. 16, 1773–1782. 10.1038/nn.3560 24162655 PMC3973738

[B19] Boeckx C. (2010). Language in Cognition: Uncovering Mental Structures and the Rules Behind Them. Malden, MA: Wiley-Blackwell.

[B20] Boeckx C. (2013). Biolinguistics: forays into human cognitive biology. J. Anthropol. Sci. 91, 63–89. 10.4436/jass.91009 24038628

[B21] Boeckx C. Benítez-Burraco A. (2014a). The shape of the human language-ready brain. Front. Psychol. 5:282. 10.3389/fpsyg.2014.00282 24772099 PMC3983487

[B22] Boeckx C. Benítez-Burraco A. (2014b). Globularity and language-readiness: generating new predictions by expanding the set of genes of interest. Front. Psychol. 5:1324. 10.3389/fpsyg.2014.01324 25505436 PMC4243498

[B23] Böhne A. Brunet F. Galiana-Arnoux D. Schultheis C. Volff J. N. (2008). Transposable elements as drivers of genomic and biological diversity in vertebrates. Chromosome Res. 16, 203–215. 10.1007/s10577-007-1202-6 18293113

[B24] Borre Y. E. Moloney R. D. Clarke G. Dinan T. G. Cryan J. F. (2014). The impact of microbiota on brain and behavior: mechanisms and therapeutic potential. Adv. Exp. Med. Biol. 817, 373–403. 10.1007/978-1-4939-0897-4_17 24997043

[B25] Borsini A. Zunszain P. A. Thuret S. Pariante C. M. (2015). The role of inflammatory cytokines as key modulators of neurogenesis. Trends Neurosci. 38, 145–157. 10.1016/j.tins.2014.12.006 25579391

[B26] Buzsáki G. Watson B. O. (2012). Brain rhythms and neural syntax: implications for efficient coding of cognitive content and neuropsychiatric disease. Dialogues Clin. Neurosci. 14, 345–367. 23393413 10.31887/DCNS.2012.14.4/gbuzsaki PMC3553572

[B27] Buzsáki G. Logothetis N. Singer W. (2013). Scaling brain size, keeping timing: evolutionary preservation of brain rhythms. Neuron 80, 751–764. 10.1016/j.neuron.2013.10.002 24183025 PMC4009705

[B28] Cargill R. Kohama S. G. Struve J. Su W. Banine F. Witkowski E.. (2012). Astrocytes in aged nonhuman primate brain gray matter synthesize excess hyaluronan. Neurobiol. Aging 33, 830.e13–830.e24. 10.1016/j.neurobiolaging.2011.07.006 21872361 PMC3227765

[B29] Chance S. A. Crow T. J. (2007). Distinctively human: cerebral lateralisation and language in Homo sapiens. J. Anthropol. Sci. 85, 83–100.

[B30] Chen W. V. Maniatis T. (2013). Clustered protocadherins. Development 140, 3297–3302. 10.1242/dev.090621 23900538 PMC3737714

[B31] Cho H. Proll S. C. Szretter K. J. Katze M. G. Gale M. Jr. Diamond M. S. (2013). Differential innate immune response programs in neuronal subtypes determine susceptibility to infection in the brain by positive-stranded RNA viruses. Nat. Med. 19, 458–464. 10.1038/nm.3108 23455712 PMC3618596

[B180] Chomsky N. (1995). The Minimalist Program. Cambridge: MIT Press.

[B32] Crisp A. Boschetti C. Perry M. Tunnacliffe A. Micklem G. (2015). Expression of multiple horizontally acquired genes is a hallmark of both vertebrate and invertebrate genomes. Genome Biol. 16:50. 10.1186/s13059-015-0607-3 25785303 PMC4358723

[B33] Crow T. J. (2008). The ‘big bang’ theory of the origin of psychosis and the faculty of language. Schizophr. Res. 102, 31–52. 10.1016/j.schres.2008.03.010 18502103

[B34] Cryan J. F. Dinan T. G. (2012). Mind-altering microorganisms: the impact of the gut microbiota on brain and behaviour. Nat. Rev. Neurosci. 13, 701–712. 10.1038/nrn3346 22968153

[B35] Desbonnet L. Clarke G. Shanahan F. Dinan T. G. Cryan J. F. (2014). Microbiota is essential for social development in the mouse. Mol. Psychiatry 19, 146–148. 10.1038/mp.2013.65 23689536 PMC3903109

[B36] Desplats P. Dumaop W. Smith D. Adame A. Everall I. Letendre S.. (2013). Molecular and pathologic insights from latent HIV-1 infection in the human brain. Neurology 80, 1415–1423. 10.1212/WNL.0b013e31828c2e9e 23486877 PMC3662272

[B37] Devanna P. Middelbeek J. Vernes S. C. (2014). FOXP2 drives neuronal differentiation by interacting with retinoic acid signaling pathways. Front. Cell. Neurosci. 8:305. 10.3389/fncel.2014.00305 25309332 PMC4176457

[B38] Dever S. M. Xu R. Fitting S. Knapp P. E. Hauser K. F. (2012). Differential expression and HIV-1 regulation of μ-opioid receptor splice variants across human central nervous system cell types. J. Neurovirol. 18, 181–190. 10.1007/s13365-012-0096-z 22528479 PMC3731452

[B39] Dibbens L. M. Tarpey P. S. Hynes K. Bayly M. A. Scheffer I. E. Smith R.. (2008). X-linked protocadherin 19 mutations cause female-limited epilepsy and cognitive impairment. Nat. Genet. 40, 776–781. 10.1038/ng.149 18469813 PMC2756413

[B40] Diem O. Schäffner M. Seifarth W. Leib-Mösch C. (2012). Influence of antipsychotic drugs on human endogenous retrovirus (HERV) transcription in brain cells. PLoS One 7:e30054. 10.1371/journal.pone.0030054 22253875 PMC3256206

[B41] Dong E. Gavin D. P. Chen Y. Davis J. (2012). Upregulation of TET1 and downregulation of APOBEC3A and APOBEC3C in the parietal cortex of psychotic patients. Transl. Psychiatry 2:e159. 10.1038/tp.2012.86 22948384 PMC3565208

[B42] Drozak J. Veiga-da-Cunha M. Vertommen D. Stroobant V. Van Schaftingen E. (2010). Molecular identification of carnosine synthase as ATP-grasp domain-containing protein 1 (ATPGD1). J. Biol. Chem. 285, 9346–9356. 10.1074/jbc.M109.095505 20097752 PMC2843183

[B43] Eggleton P. Tenner A. J. Reid K. B. (2000). C1q receptors. Clin. Exp. Immunol. 120, 406–412. 10.1046/j.1365-2249.2000.01218.x 10844516 PMC1905565

[B44] Enard W. Khaitovich P. Klose J. Zöllner S. Heissig F. Giavalisco P.. (2002). Intra- and interspecific variation in primate gene expression patterns. Science 296, 340–343. 10.1126/science.1068996 11951044

[B47] Feynman R. P. (1960). There’s plenty of room at the bottom. EandS 23, 22–36.

[B48] Fitzpatrick B. M. (2014). Symbiote transmission and maintenance of extra-genomic associations. Front. Microbiol. 5:46. 10.3389/fmicb.2014.00046 24605109 PMC3932413

[B49] Fodor J. (1975). The Language of Thought. New York, NY: Crowell & Moring LLP.

[B50] Fodor J. (1994). The Elm and the Expert: Mentalese and Its Semantics. Cambridge, MA: MIT Press.

[B51] Fodor J. (1998). Concepts: Where Cognitive Science Went Wrong. New York: Oxford University Press.

[B52] Frank O. Giehl M. Zheng C. Hehlmann R. Leib-Mösch C. Seifarth W. (2005). Human endogenous retrovirus expression profiles in samples from brains of patients with schizophrenia and bipolar disorders. J. Virol. 79, 10890–10901. 10.1128/jvi.79.17.10890-10901.2005 16103141 PMC1193590

[B53] Franzenburg S. Walter J. Künzel S. Wang J. Baines J. F. Bosch T. C. G.. (2013). Distinct antimicrobial peptide expression determines host species-specific bacterial associations. Proc. Natl. Acad. Sci. U S A 110, E3730–E3738. 10.1073/pnas.1304960110 24003149 PMC3785777

[B54] Fruntes V. Limosin F. (2008). Schizophrenia and viral infection during neurodevelopment: a pathogenesis model? Med. Sci. Monit. 14, RA71–RA77. 18509285

[B55] Gallistel C. R. King A. P. (2009). Memory and the Computational Brain: Why Cognitive Science Will Transform Neuroscience. New York: Wiley/Blackwell.

[B181] Garay P. A. McAllister A. K. (2010). Novel roles for immune molecules in neural development: implications for neurodevelopmental disorders. Front. Synaptic. Neurosci. 2:136 10.3389/fnsyn.2010.00136 21423522 PMC3059681

[B56] Garbett K. A. Hsiao E. Y. Kálmán S. Patterson P. H. Mirnics K. (2012). Effects of maternal immune activation on gene expression patterns in the fetal brain. Transl. Psychiatry 2:e98. 10.1038/tp.2012.24 22832908 PMC3337077

[B57] Ge J. Li L. Jin Q. Liu Y. C. Zhao L. Song H. H. (2014). Functional IRGM polymorphism is associated with language impairment in glioma and upregulates cytokine expressions. Tumour Biol. 35, 8343–8348. 10.1007/s13277-014-2091-x 24859836

[B58] Giraud A.-L. Poeppel D. (2012). Cortical oscillations and speech processing: emerging computational principles and operations. Nat. Neurosci. 15, 511–517. 10.1038/nn.3063 22426255 PMC4461038

[B186] Glaser B. Mumme D. L. Blasey C. Morris M. A. Dahoun S. P. Antonarakis S. E.. (2002). Language skills in children with velocardiofacial syndrome (deletion 22q11.2). J. Pediatr. 140, 753–758. 10.1067/mpd.2002.124774 12072882

[B59] Gokhman D. Lavi E. Prüfer K. Fraga M. F. Riancho J. A. Kelso J.. (2014). Reconstructing the DNA methylation maps of the neandertal and the denisovan. Science 344, 523–527. 10.1126/science.1250368 24786081

[B60] Graham S. A. Fisher S. E. (2013). Decoding the genetics of speech and language. Curr. Opin. Neurobiol. 23, 43–51. 10.1016/j.conb.2012.11.006 23228431

[B61] Green R. E. Krause J. Briggs A. W. Maricic T. Stenzel U. Kircher M.. (2010). A draft sequence of the Neandertal genome. Science 328, 710–722. 10.1126/science.1188021 20448178 PMC5100745

[B62] Hancks D. C. Kazazian H. H. Jr. (2012). Active human retrotransposons: variation and disease. Curr. Opin. Genet. Dev. 22, 191–203. 10.1016/j.gde.2012.02.006 22406018 PMC3376660

[B63] Hansen S. M. Berezin V. Bock E. (2008). Signaling mechanisms of neurite outgrowth induced by the cell adhesion molecules NCAM and N-cadherin. Cell. Mol. Life Sci. 65, 3809–3821. 10.1007/s00018-008-8290-0 18791849 PMC11131707

[B65] Hauser M. D. Chomsky N. Fitch W. T. (2002). The faculty of language: what is it, who has it and how did it evolve? Science 298, 1569–1579. 10.1126/science.298.5598.1569 12446899

[B66] Heiber M. Docherty J. M. Shah G. Nguyen T. Cheng R. Heng H. H. Q.. (1995). Isolation of three novel human genes encoding G protein-coupled receptors. DNA Cell Biol. 14, 25–35. 10.1089/dna.1995.14.25 7832990

[B68] Hirabayashi T. Yagi T. (2014). Protocadherins in neurological diseases. Adv. Neurobiol. 8, 293–314. 10.1007/978-1-4614-8090-7_13 25300142

[B69] Hirayama T. Yagi T. (2013). Clustered protocadherins and neuronal diversity. Prog. Mol. Biol. Transl. Sci. 116, 145–167. 10.1016/B978-0-12-394311-8.00007-8 23481194

[B70] Horie M. Honda T. Suzuki Y. Kobayashi Y. Daito T. Oshida T.. (2010). Endogenous non-retroviral RNA virus elements in mammalian genomes. Nature 463, 84–87. 10.1038/nature08695 20054395 PMC2818285

[B71] Hranilovic D. Bujas-Petkovic Z. Vragovic R. Vuk T. Hock K. Jernej B. (2007). Hyperserotonemia in adults with autistic disorder. J. Autism. Dev. Disord. 37, 1934–1940. 10.1007/s10803-006-0324-6 17165147

[B72] Hsiao E. Y. McBride S. W. Hsien S. Sharon G. Hyde E. R. McCue T.. (2013). Microbiota modulate behavioral and physiological abnormalities associated with neurodevelopmental disorders. Cell 155, 1451–1463. 10.1016/j.cell.2013.11.024 24315484 PMC3897394

[B73] Ishida Y. Zhao K. Greenwood A. D. Roca A. L. (2015). Proliferation of endogenous retroviruses in the early stages of a host germ line invasion. Mol. Biol. Evol. 32, 109–120. 10.1093/molbev/msu275 25261407 PMC4271524

[B74] Ishigami A. Ohsawa T. Hiratsuka M. Taguchi H. Kobayashi S. Saito Y.. (2005). Abnormal accumulation of citrullinated proteins catalyzed by peptidylarginine deiminase in hippocampal extracts from patients with Alzheimer’s disease. J. Neurosci. Res. 80, 120–128. 10.1002/jnr.20431 15704193

[B75] Ju X. D. Guo Y. Wang N. N. Huang Y. Lai M. M. Zhai Y. H.. (2014). Both Myosin-10 isoforms are required for radial neuronal migration in the developing cerebral cortex. Cereb. Cortex 24, 1259–1568. 10.1093/cercor/bhs407 23300110

[B76] Kalscheuer V. M. Tao J. Donnelly A. Hollway G. Schwinger E. Kubart S.. (2003). Disruption of the serine/threonine kinase 9 gene causes severe X-linked infantile spasms and mental retardation. Am. J. Hum. Genet. 72, 1401–1411. 10.1086/375538 12736870 PMC1180301

[B77] Karim S. Mirza Z. Kamal M. A. Abuzenadah A. M. Azhar E. I. Al-Q14htani M. H.. (2014). The role of viruses in neurodegenerative and neurobehavioral diseases. CNS Neurol. Disord. Drug Targets 13, 1213–1223. 10.2174/187152731307141015122638 25230220

[B78] Khaitovich P. Enard W. Lachmann M. Pääbo S. (2006). Evolution of primate gene expression. Nat. Rev. Genet. 7, 693–702. 10.1038/nrg1940 16921347

[B191] Kidwell M. G. (2002). Transposable elements and the evolution of genome size in eukaryotes. Genetica 115, 49–63. 12188048 10.1023/a:1016072014259

[B79] Kokaia Z. Martino G. Schwartz M. Lindvall O. (2012). Cross-talk between neural stem cells and immune cells: the key to better brain repair? Nat. Neurosci. 15, 1078–1087. 10.1038/nn.3163 22837038

[B80] Koonin E. V. Wolf Y. I. (2012). Evolution of microbes and viruses: a paradigm shift in evolutionary biology? Front. Cell. Infect. Microbiol. 2:119. 10.3389/fcimb.2012.00119 22993722 PMC3440604

[B81] Kuhlwilm M. Davierwala A. Pääbo S. (2013). Identification of putative target genes of the transcription factor RUNX2. PLoS One 8:e83218. 10.1371/journal.pone.0083218 24349465 PMC3861491

[B82] Kurahashi H. Inagaki H. Hosoba E. Kato T. Ohye T. Kogo H.. (2007). Molecular cloning of a translocation breakpoint hotspot in 22q11. Genome Res. 17, 461–469. 10.1101/gr.5769507 17267815 PMC1832093

[B83] Le Dantec C. Vallet S. Brooks W. H. Renaudineau Y. (2015). Human endogenous retrovirus group e and its involvement in diseases. Viruses 7, 1238–1257. 10.3390/v7031238 25785516 PMC4379568

[B84] Li C. Ito H. Fujita K. Shiwaku H. Qi Y. Tagawa K.. (2013). Sox2 transcriptionally regulates PQ15P1, an intellectual disability-microcephaly causative gene, in neural stem progenitor cells. PLoS One 8:e68627. 10.1371/journal.pone.0068627 23874697 PMC3713010

[B185] Lionel A. C. Tammimies K. Vaags A. K. Rosenfeld J. A. Ahn J. W. Merico D. (2014). Disruption of the ASTN2/TRIM32 locus at 9q33.1 is a risk factor in males for autism spectrum disorders, ADHD and other neurodevelopmental phenotypes. Hum. Mol. Genet. 23, 2752–2768. 10.1093/hmg/ddt669 24381304 PMC3990173

[B85] Litman G. W. Rast J. P. Fugmann S. D. (2010). The origins of vertebrate adaptive immunity. Nat. Rev. Immunol. 10, 543–553. 10.1038/nri2807 20651744 PMC2919748

[B86] Liu H. Fu Y. Li B. Yu X. Xie J. Cheng J.. (2011). Widespread horizontal gene transfer from circular single-stranded DNA viruses to eukaryotic genomes. BMC Evol. Biol. 11:276. 10.1186/1471-2148-11-276 21943216 PMC3198968

[B88] Liu X. Somel M. Tang L. Yan Z. Jiang X. Guo S.. (2012a). Extension of cortical synaptic development distinguishes humans from chimpanzees and macaques. Genome Res. 22, 611–622. 10.1101/gr.127324.111 22300767 PMC3317144

[B87] Liu H. Wang X. Wang H.-D. Wu J. Ren J. Meng L.. (2012b). Escherichia coli noncoding RNAs can affect gene expression and physiology of Caenorhabditis elegans. Nat. Commun. 3:1073. 10.1038/ncomms2071 23011127 PMC3658002

[B89] Lombardo M. P. (2008). Access to mutualistic endosymbiotic microbes: an underappreciated benefit of group living. Behav. Ecol. Sociobiol. 62, 479–497. 10.1007/s00265-007-0428-9

[B90] MacFabe D. F. Cain N. E. Boon F. Ossenkopp K.-P. Cain D. P. (2011). Effects of the enteric bacterial metabolic product propionic acid on object-directed behavior, social behavior, cognition and neuroinflammation in adolescent rats: relevance to autism spectrum disorder. Behav. Brain Res. 217, 47–54. 10.1016/j.bbr.2010.10.005 20937326

[B91] Maness P. F. Schachner M. (2007). Neural recognition molecules of the immunoglobulin superfamily: signaling transducers of axon guidance and neuronal migration. Nat. Neurosci. 10, 19–26. 10.1038/nn1827 17189949

[B92] Marchese A. Cheng R. Lee M. C. Porter C. A. Heiber M. Goodman M.. (1994). Mapping studies of two G protein-coupled receptor genes: an amino acid difference may confer a functional variation between a human and rodent receptor. Biochem. Biophys. Res. Commun. 205, 1952–1958. 10.1006/bbrc.1994.2899 7811287

[B93] Maricic T. Günther V. Georgiev O. Gehre S. Curlin M. Schreiweis C.. (2013). A recent evolutionary change affects a regulatory element in the human FOXP2 gene. Mol. Biol. Evol. 30, 844–852. 10.1093/molbev/mss271 23197593

[B94] Marques F. Brito M. J. Conde M. Pinto M. Moreira A. (2014). Autism spectrum disorder secondary to enterovirus encephalitis. J. Child Neurol. 29, 708–714. 10.1177/0883073813508314 24782421

[B95] Mastronardi F. G. Noor A. Wood D. D. Paton T. Moscarello M. A. (2007). Peptidyl argininedeiminase 2 CpG island in multiple sclerosis white matter is hypomethylated. J. Neurosci. Res. 85, 2006–2016. 10.1002/jnr.21329 17469138

[B96] McLean C. Y. Reno P. L. Pollen A. A. Bassan A. I. Capellini T. D. Guenther C.. (2011). Human-specific loss of regulatory DNA and the evolution of human-specific traits. Nature 471, 216–219. 10.1038/nature09774 21390129 PMC3071156

[B97] Meyer M. Kircher M. Gansauge M. T. Li H. Racimo F. Mallick S.. (2012). A high-coverage genome sequence from an archaic Denisovan individual. Science 338, 222–226. 10.1126/science.1224344 22936568 PMC3617501

[B98] Ming X. Stein T. P. Barnes V. Rhodes N. Guo L. (2012). Metabolic perturbance in autism spectrum disorders: a metabolomics study. J. Proteome Res. 11, 5856–5862. 10.1021/pr300910n 23106572

[B99] Mohamed H. A. Mosier D. R. Zou L. L. Siklós L. Alexianu M. E. Engelhardt J. I.. (2002). Immunoglobulin Fc gamma receptor promotes immunoglobulin uptake, immunoglobulin-mediated calcium increase and neurotransmitter release in motor neurons. J. Neurosci. Res. 69, 110–116. 10.1002/jnr.10271 12111822

[B100] Montiel-Castro A. J. Báez-Yáñez M. G. Pacheco-López G. (2014). Social neuroeconomics: the influence of microbiota in partner-choice and sociality. Curr. Pharm. Des. 20, 4774–4783. 10.2174/1381612820666140130210631 24588821

[B101] Montiel-Castro A. J. González-Cervantes R. M. Bravo-Ruiseco G. Pacheco-López G. (2013). The microbiota-gut-brain axis: neurobehavioral correlates, health and sociality. Front. Integr. Neurosci. 7:70. 10.3389/fnint.2013.00070 24109440 PMC3791857

[B102] Moreno-De-Luca A. Helmers S. L. Mao H. Burns T. G. Melton A. M. Schmidt K. R.. (2011). Adaptor protein complex-4 (AP-4) deficiency causes a novel autosomal recessive cerebral palsy syndrome with microcephaly and intellectual disability. J. Med. Genet. 48, 141–144. 10.1136/jmg.2010.082263 20972249 PMC3150730

[B103] Moretti R. Pansiot J. Bettati D. Strazielle N. Ghersi-Egea J. F. Damante G.. (2015). Blood-brain barrier dysfunction in disorders of the developing brain. Front. Neurosci. 9:40. 10.3389/fnins.2015.00040 25741233 PMC4330788

[B104] Moskovitz J. Walss-Bass C. Cruz D. A. Thompson P. M. Bortolato M. (2014). Methionine sulfoxide reductase regulates brain catechol-O-methyl transferase activity. Int. J. Neuropsychopharmacol. 17, 1707–1713. 10.1017/s1461145714000467 24735585 PMC4398661

[B105] Muotri A. R. Marchetto M. C. Coufal N. G. Oefner R. Yeo G. Nakashima K.. (2010). L1 retrotransposition in neurons is modulated by MeCP2. Nature 468, 443–446. 10.1038/nature09544 21085180 PMC3059197

[B106] Nakahara J. Tan-Takeuchi K. Seiwa C. Gotoh M. Kaifu T. Ujike A.. (2003). Signaling via immunoglobulin Fc receptors induces oligodendrocyte precursor cell differentiation. Dev. Cell 4, 841–852. 10.1016/s1534-5807(03)00155-2 12791269

[B107] Nakamura K. Hirai H. Torashima T. Miyazaki T. Tsurui H. Xiu Y.. (2007). CD3 and immunoglobulin G Fc receptor regulate cerebellar functions. Mol. Cell. Biol. 27, 5128–5134. 10.1128/mcb.01072-06 17502348 PMC1951947

[B108] Neale J. H. Olszewski R. T. Zuo D. Janczura K. J. Profaci C. P. Lavin K. M.. (2011). Advances in understanding the peptide neurotransmitter NAAG and appearance of a new member of the NAAG neuropeptide family. J. Neurochem. 118, 490–498. 10.1111/j.1471-4159.2011.07338.x 21644997 PMC3137677

[B109] Nijveen H. Matus-Garcia M. van Passel M. W. (2012). Promoter reuse in prokaryotes. Mob. Genet. Elements 2, 279–281. 10.4161/mge.23195 23481313 PMC3575422

[B110] O’Mahony S. M. Clarke G. Borre Y. E. Dinan T. G. Cryan J. F. (2015). Serotonin, tryptophan metabolism and the brain-gut-microbiome axis. Behav. Brain Res. 277, 32–48. 10.1016/j.bbr.2014.07.027 25078296

[B111] O’Mahony S. M. Marchesi J. R. Scully P. Codling C. Ceolho A.-M. Quigley E. M. M.. (2009). Early life stress alters behavior, immunity and microbiota in rats: implications for irritable bowel syndrome and psychiatric illnesses. Biol. Psychiatry 65, 263–267. 10.1016/j.biopsych.2008.06.026 18723164

[B112] Okun E. Mattson M. P. Arumugam T. V. (2010). Involvement of Fc receptors in disorders of the central nervous system. Neuromolecular Med. 12, 164–178. 10.1007/s12017-009-8099-5 19844812 PMC2878892

[B113] Oren Y. Smith M. B. Johns N. I. Kaplan Zeevi M. Biran D. Ron E. Z.. (2014). Transfer of noncoding DNA drives regulatory rewiring in bacteria. Proc. Natl. Acad. Sci. U S A 111, 16112–161127. 10.1073/pnas.1413272111 25313052 PMC4234569

[B114] Pääbo S. (2014). The human condition-a molecular approach. Cell 157, 216–226. 10.1016/j.cell.2013.12.036 24679537

[B115] Paolicelli R. C. Bolasco G. Pagani F. Maggi L. Scianni M. Panzanelli P.. (2011). Synaptic pruning by microglia is necessary for normal brain development. Science 333, 1456–1458. 10.1126/science.1202529 21778362

[B116] Pascual I. Larrayoz I. M. Rodríguez I. R. (2009). Retinoic acid regulates the human methionine sulfoxide reductase A (MSRA) gene via two distinct promoters. Genomics 93, 62–71. 10.1016/j.ygeno.2008.09.002 18845237 PMC2645438

[B117] Patterson P. H. (2011). Maternal infection and immune involvement in autism. Trends Mol. Med. 17, 389–394. 10.1016/j.molmed.2011.03.001 21482187 PMC3135697

[B118] Pemberton H. N. Franklyn J. A. Boelaert K. Chan S. Y. Kim D. S. Kim C.. (2007). Separase, securin and Rad21 in neural cell growth. J. Cell. Physiol. 213, 45–53. 10.1002/jcp.21086 17450531

[B119] Perron H. Lang A. (2010). The human endogenous retrovirus link between genes and environment in multiple sclerosis and in multifactorial diseases associating neuroinflammation. Clin. Rev. Allergy Immunol. 39, 51–61. 10.1007/s12016-009-8170-x 19697163

[B120] Piattelli-Palmarini M. Uriagereka J. (2004). “The immune syntax: the evolution of the language virus,” in Variation and Universals in Biolinguistics, ed. Jenkins L. (Oxford: Elsevier), 341–377.

[B121] Piattelli-Palmarini M. Uriagereka J. (2008). Still a bridge too far? Biolinguistic questions for grounding language on brains. Phys. Life Rev. 5, 207–224. 10.1016/j.plrev.2008.07.002

[B122] Poeppel D. (2012). The maps problem and the mapping problem: two challenges for a cognitive neuroscience of speech and language. Cogn. Neuropsychol. 29, 34–55. 10.1080/02643294.2012.710600 23017085 PMC3498052

[B123] Prag S. Lepekhin E. A. Kolkova K. Hartmann-Petersen R. Kawa A. Walmod P. S.. (2002). NCAM regulates cell motility. J. Cell Sci. 115, 283–292. 11839780 10.1242/jcs.115.2.283

[B124] Prüfer K. Racimo F. Patterson N. Jay F. Sankararaman S. Sawyer S.. (2014). The complete genome sequence of a Neanderthal from the Altai Mountains. Nature 505, 43–49. 10.1038/nature12886 24352235 PMC4031459

[B125] Ransohoff R. M. Brown M. A. (2012). Innate immunity in the central nervous system. J. Clin. Invest. 122, 1164–1171. 10.1172/JCI58644 22466658 PMC3314450

[B126] Ratnayake U. Quinn T. Walker D. W. Dickinson H. (2013). Cytokines and the neurodevelopmental basis of mental illness. Front. Neurosci. 7:180. 10.3389/fnins.2013.00180 24146637 PMC3797953

[B127] Ribes V. Fraulob V. Petkovich M. Dollé P. (2007). The oxidizing enzyme CYP26a1 tightly regulates the availability of retinoic acid in the gastrulating mouse embryo to ensure proper head development and vasculogenesis. Dev. Dyn. 236, 644–653. 10.1002/dvdy.21057 17211890

[B129] Roeske T. C. Scharff C. Olson C. R. Nshdejan A. Mello C. V. (2014). Long-distance retinoid signaling in the zebra finch brain. PLoS One 9:e111722. 10.1371/journal.pone.0111722 25393898 PMC4230966

[B130] Rosca E. C. Rosca O. Simu M. Chirileanu R. D. (2012). HIV-associated neurocognitive disorders: a historical review. Neurologist 18, 64–67. 10.1097/NRL.0b013e318247bc7a 22367830

[B131] Rosenberg E. Zilber-Rosenberg I. (2014). The Hologenome Concept: Human, Animal and Plant Microbiota. New York: Springer International Publishing.

[B132] Scala E. Ariani F. Mari F. Caselli R. Pescucci C. Longo I.. (2005). CDKL5/STK9 is mutated in Rett syndrome variant with infantile spasms. J. Med. Genet. 42, 103–107. 10.1136/jmg.2004.026237 15689447 PMC1735977

[B133] Schellekens H. Clarke G. Jeffery I. B. Dinan T. G. Cryan J. F. (2012). Dynamic 5-HT2C receptor editing in a mouse model of obesity. PLoS One 7:e32266. 10.1371/journal.pone.0032266 22448217 PMC3308946

[B134] Schmucker D. Chen B. (2009). Dscam and DSCAM: complex genes in simple animals, complex animals yet simple genes. Genes Dev. 23, 147–156. 10.1101/gad.1752909 19171779

[B135] Seifarth W. Frank O. Zeilfelder U. Spiess B. Greenwood A. D. Hehlmann R.. (2005). Comprehensive analysis of human endogenous retrovirus transcriptional activity in human tissues with a retrovirus-specific microarray. J. Virol. 79, 341–352. 10.1128/jvi.79.1.341-352.2005 15596828 PMC538696

[B136] Shimizu H. Iwayama Y. Yamada K. Toyota T. Minabe Y. Nakamura K.. (2006). Genetic and expression analyses of the STOP (MAP6) gene in schizophrenia. Schizophr. Res. 84, 244–252. 10.1016/j.schres.2006.03.017 16624526

[B137] Shimizu N. Soda Y. Kanbe K. Liu H. Y. Jinno A. Kitamura T.. (1999). An orphan G protein-coupled receptor, GPR1, acts as a coreceptor to allow replication of human immunodeficiency virus types 1 and 2 in brain-derived cells. J. Virol. 73, 5231–5239. 10233994 10.1128/jvi.73.6.5231-5239.1999 PMC112576

[B138] Shirazi J. Shah S. Sagar D. Nonnemacher M. R. Wigdahl B. Khan Z. K.. (2013). Epigenetics, drugs of abuse and the retroviral promoter. J. Neuroimmune Pharmacol. 8, 1181–1196. 10.1007/s11481-013-9508-y 24218017 PMC3878082

[B139] Siddique A. Buisine N. Chalmers R. (2011). The transposon-like Correia elements encode numerous strong promoters and provide a potential new mechanism for phase variation in the meningococcus. PLoS Genet. 7:e1001277. 10.1371/journal.pgen.1001277 21283790 PMC3024310

[B140] Sikela J. M. (2006). The jewels of our genome: the search for the genomic changes underlying the evolutionarily unique capacities of the human brain”. PLoS Genet. 2:e80. 10.1371/journal.pgen.0020080 16733552 PMC1464830

[B141] Sotomayor M. Gaudet R. Corey D. P. (2014). Sorting out a promiscuous superfamily: towards cadherin connectomics. Trends Cell Biol. 24, 524–536. 10.1016/j.tcb.2014.03.007 24794279 PMC4294768

[B142] Spelke E. S. (1994). Initial knowledge: six suggestions. Cognition 50, 431–445. 10.1016/0010-0277(94)90039-6 8039373

[B143] Spelke E. S. (2000). Core knowledge. Am. Psychol. 55, 1233–1243. 10.1037/0003-066X.55.11.1233 11280937

[B144] Spelke E. S. (2003). “What makes us smart?,” in Language in Mind, eds Gentner D. Goldin-Meadow S. (Cambridge, MA: MIT Press), 277–311.

[B145] Spiteri E. Konopka G. Coppola G. Bomar J. Oldham M. Ou J.. (2007). Identification of the transcriptional targets of FOXP2, a gene linked to speech and language, in developing human brain. . Am. J. Hum. Genet. 81, 1144–1157. 10.1086/522237 17999357 PMC2276350

[B147] Stilling R. M. Bordenstein S. R. Dinan T. G. Cryan J. F. (2014b). Friends with social benefits: host-microbe interactions as a driver of brain evolution and development? Front. Cell. Infect. Microbiol. 4:147. 10.3389/fcimb.2014.00147 25401092 PMC4212686

[B146] Stilling R. M. Dinan T. G. Cryan J. F. (2014a). Microbial genes, brain and behaviour - epigenetic regulation of the gut-brain axis. Genes Brain Behav. 13, 69–86. 10.1111/gbb.12109 24286462

[B148] Suntsova M. Gogvadze E. V. Salozhin S. Gaifullin N. Eroshkin F. Dmitriev S. E.. (2013). Human-specific endogenous retroviral insert serves as an enhancer for the schizophrenia-linked gene PRODH. Proc. Natl. Acad. Sci. U S A 110, 19472–19477. 10.1073/pnas.1318172110 24218577 PMC3845128

[B149] Swillen A. Devriendt K. Legius E. Prinzie P. Vogels A. Ghesquiere P.. (1999). The behavioural phenotype in velo-cardio-facial syndrome (VCFS): from infancy to adolescence. Genet. Couns. 10, 79–88. 10191433

[B150] Syvanen M. (2012). Evolutionary implications of horizontal gene transfer. Annu. Rev. Genet. 46, 341–358. 10.1146/annurev-genet-110711-155529 22934638

[B182] Szklarczyk D. Franceschini A. Wyder S. Forslund K. Heller D. Huerta-Cepas J. (2015). STRING v10: protein-protein interaction networks, integrated over the tree of life. Nucleic Acids Res. 43, D447–D452. 10.1093/nar/gku1003 25352553 PMC4383874

[B151] Tao J. Van Esch H. Hagedorn-Greiwe M. Hoffmann K. Moser B. Raynaud M.. (2004). Mutations in the X-linked cyclin-dependent kinase-like 5 (CDKL5/STK9) gene are associated with severe neurodevelopmental retardation. Am. J. Hum. Genet. 75, 1149–1154. 10.1086/426460 15499549 PMC1182152

[B152] Thomas C. A. Paquola A. C. Muotri A. R. (2012). LINE-1 retrotransposition in the nervous system. Annu. Rev. Cell Dev. Biol. 28, 555–573. 10.1146/annurev-cellbio-101011-155822 23057747

[B153] Tomasello M. (2009). The Cultural Origins of Human Cognition. Cambridge: Harvard University Press.

[B154] Tomasello M. (2014). A Natural History of Human Thinking. Cambridge: Harvard University Press.

[B155] Tsai N. P. Wilkerson J. R. Guo W. Maksimova M. A. DeMartino G. N. Cowan C. W.. (2012). Multiple autism-linked genes mediate synapse elimination via proteasomal degradation of a synaptic scaffold PSD-95. Cell 151, 1581–1594. 10.1016/j.cell.2012.11.040 23260144 PMC3530171

[B156] Uehara M. Yashiro K. Mamiya S. Nishino J. Chambon P. Dolle P.. (2007). CYP26A1 and CYP26C1 cooperatively regulate anterior-posterior patterning of the developing brain and the production of migratory cranial neural crest cells in the mouse. Dev. Biol. 302, 399–411. 10.1016/j.ydbio.2006.09.045 17067568

[B157] Vallender E. J. Mekel-Bobrov N. Lahn B. T. (2008). Genetic basis of human brain evolution. Trends Neurosci. 31, 637–644. 10.1016/j.tins.2008.08.010 18848363 PMC2715140

[B183] van der Meer P. Ulrich A. M. Gonźalez-Scarano F. Lavi E. (2000). Immunohistochemical analysis of CCR2, CCR3, CCR5, and CXCR4 in the human brain: potential mechanisms for HIV dementia. Exp. Mol. Pathol. 69, 192–201. 10.1006/exmp.2000.2336 11115360

[B158] Varki A. Geschwind D. H. Eichler E. E. (2008). Explaining human uniqueness: genome interactions with environment, behaviour and culture. Nat. Rev. Genet. 9, 749–763. 10.1038/nrg2428 18802414 PMC2756412

[B159] Vawter M. P. Usen N. Thatcher L. Ladenheim B. Zhang P. VanderPutten D. M.. (2001). Characterization of human cleaved N-CAM and association with schizophrenia. Exp. Neurol. 172, 29–46. 10.1006/exnr.2001.7790 11681838

[B160] Vernes S. C. Oliver P. L. Spiteri E. Lockstone H. E. Puliyadi R. Taylor J. M.. (2011). Foxp2 regulates gene networks implicated in neurite outgrowth in the developing brain. PLoS Genet. 7:e1002145. 10.1371/journal.pgen.1002145 21765815 PMC3131290

[B161] Virgin H. W. (2014). The virome in mammalian physiology and disease. Cell 157, 142–150. 10.1016/j.cell.2014.02.032 24679532 PMC3977141

[B162] Volle J. Brocard J. Saoud M. Gory-Faure S. Brunelin J. Andrieux A.. (2013). Reduced expression of STOP/MAP6 in mice leads to cognitive deficits. Schizophr. Bull. 39, 969–978. 10.1093/schbul/sbs113 23002183 PMC3756782

[B163] Wall R. Cryan J. F. Ross R. P. Fitzgerald G. F. Dinan T. G. Stanton C. (2014). Bacterial neuroactive compounds produced by psychobiotics. Adv. Exp. Med. Biol. 817, 221–239. 10.1007/978-1-4939-0897-4_10 24997036

[B164] Wang K. S. Tonarelli S. Luo X. Wang L. Su B. Zuo L.. (2015a). Polymorphisms within ASTN2 gene are associated with age at onset of Alzheimer’s disease. J. Neural Transm. (Vienna) 122, 701-708. 10.1007/s00702-014-1306-z 25410587

[B165] Wang T. Jiang X. Chen G. Xu J. (2015b). Interaction of amyotrophic lateral sclerosis/frontotemporal lobar degeneration-associated fused-in-sarcoma with proteins involved in metabolic and protein degradation pathways. Neurobiol. Aging. 36, 527–535. 10.1016/j.neurobiolaging.2014.07.044 25192599

[B190] Wagner E. Frank M. M. (2010). Therapeutic potential of complement modulation. Nat. Rev. Drug Discov. 9, 43–56. 10.1038/nrd3011 19960015

[B166] Weckselblatt B. Rudd M. K. (2015). Human structural variation: mechanisms of chromosome rearrangements. Trends Genet. 31, 587–599. 10.1016/j.tig.2015.05.010 26209074 PMC4600437

[B167] Williams N. A. Close J. P. Giouzeli M. Crow T. J. (2006). Accelerated evolution of Protocadherin11X/Y: a candidate gene-pair for cerebral asymmetry and language. Am. J. Med. Genet. B Neuropsychiatr. Genet. 141B, 623–633. 10.1002/ajmg.b.30357 16874762

[B168] Williamson L. L. Bilbo S. D. (2013). Chemokines and the hippocampus: a new perspective on hippocampal plasticity and vulnerability. Brain Behav. Immun. 30, 186–194. 10.1016/j.bbi.2013.01.077 23376170

[B169] Wilson P. M. Fryer R. H. Fang Y. Hatten M. E. (2010). Astn2, a novel member of the astrotactin gene family, regulates the trafficking of ASTN1 during glial-guided neuronal migration. J. Neurosci. 30, 8529–8540. 10.1523/JNEUROSCI.0032-10.2010 20573900 PMC2905051

[B170] Wu Y. Sheng W. Chen L. Dong H. Lee V. Lu F.. (2004). Versican V1 isoform induces neuronal differentiation and promotes neurite outgrowth. Mol. Biol. Cell 15, 2093–2104. 10.1091/mbc.e03-09-0667 14978219 PMC404007

[B171] Xiang Y. Y. Dong H. Wan Y. Li J. Yee A. Yang B. B.. (2006). Versican G3 domain regulates neurite growth and synaptic transmission of hippocampal neurons by activation of epidermal growth factor receptor. J. Biol. Chem. 281, 19358–19368. 10.1074/jbc.m512980200 16648628

[B172] Yagi T. (2012). Molecular codes for neuronal individuality and cell assembly in the brain. Front. Mol. Neurosci. 5:45. 10.3389/fnmol.2012.00045 22518100 PMC3324988

[B173] Yagi T. (2013). Genetic basis of neuronal individuality in the mammalian brain. J. Neurogenet. 27, 97–105. 10.3109/01677063.2013.801969 23808929 PMC3852966

[B174] Yi J. M. Kim H. M. Kim H. S. (2006). Human endogenous retrovirus HERV-H family in human tissues and cancer cells: expression, identification and phylogeny. Cancer Lett. 231, 228–239. 10.1016/j.canlet.2005.02.001 16399224

[B175] Yoon D. S. Kim Y. H. Lee S. Lee K. M. Park K. H. Jang Y.. (2014). Interleukin-6 induces the lineage commitment of bone marrow-derived mesenchymal multipotent cells through down-regulation of Sox2 by osteogenic transcription factors. FASEB J. 28, 3273–3286. 10.1096/fj.13-248567 24719354

[B177] Zheng G. Dahl J. A. Niu Y. Fu Y. Klungland A. Yang Y.-G.. (2013). Sprouts of RNA epigenetics: the discovery of mammalian RNA demethylases. RNA Biol. 10, 915–918. 10.4161/rna.24711 23619745 PMC3904589

[B178] Ziemssen T. Kern S. (2007). Psychoneuroimmunology-cross-talk between the immune and nervous systems. J. Neurol. 254, 8–11. 10.1007/s00415-007-2003-8 17503136

